# Willingness to pay for small solar powered bed net fans: results of a Becker–DeGroot–Marschak auction in Ghana

**DOI:** 10.1186/s12936-017-1965-y

**Published:** 2017-08-07

**Authors:** Joshua O. Yukich, Olivier J. T. Briët, Collins K. Ahorlu, Peter Nardini, Joseph Keating

**Affiliations:** 1 Center for Applied Malaria Research and Evaluation, Tulane University School of Public Health and Tropical Medicine, 1440 Canal St. #8317, New Orleans, LA 70112 USA; 2 Swiss Tropical and Public Health Institute, Socinstrasse 57, P.O. Box CH-4002 Basel, Switzerland; 3 University of Basel, Basel, Switzerland; 4 Noguchi Memorial Institute for Medical Research, University of Ghana, Accra, Ghana; 5 Green World Health Net, Santa Fe, NM USA

**Keywords:** Malaria, Bednets, Fans, Experimental auction, Willingness to pay

## Abstract

**Background and methods:**

Long-lasting insecticidal nets (LLINs) are one of the main interventions recommended by the World Health Organization for malaria vector control. LLINs are ineffective if they are not being used. Subsequent to the completion of a cluster randomized cross over trial conducted in rural Greater Accra where participants were provided with the ‘Bɔkɔɔ System’—a set of solar powered net fan and light consoles with a solar panel and battery—or alternative household water filters, all trial participants were invited to participate in a Becker–DeGroot–Marschak auction to determine the mean willingness to pay (WTP) for the fan and light consoles and to estimate the demand curve for the units.

**Results, discussion and conclusions:**

Results demonstraed a mean WTP of approximately 55 Cedis (~13 USD). Demand results suggested that at a price which would support full manufacturing cost recovery, a majority of households in the area would be willing to purchase at least one such unit.

## Background

Long-lasting insecticidal nets (LLINs) are one of the main interventions recommended by the World Health Organization (WHO) for malaria vector control. These tools have nearly halved the burden of malaria in sub-Saharan Africa since their introduction at large scales in the early 2000s [1]. LLINs are, however, ineffective if they are not being used. Furthermore, population-based surveys demonstrate that net use among those owning an LLIN varies widely among countries [2]. The main reasons for net non-use include discomfort due to heat, social factors related to absence or disruption of sleeping arrangements and a perceived low density of mosquitoes [3]. These factors may also interact to limit use. It is well documented that the inside of a mosquito net may feel stuffy during hot weather because the nets reduce ventilation [4] and it has been reported that mosquito net use varies depending on the level of nuisance biting mosquito presence and temperature [5]. Behaviour change communication (BCC) has the potential to improve net usage during warm weather, but modifications to nets that increase the comfort level under the net could compliment or enhance the effectiveness of educational or BCC interventions [3]. While net manufacturers can increase mesh size to allow increased ventilation, they are also limited by mesh size specifications in international tenders as well as by perceived increased penetration by biting insects with larger mesh size [6]. After experimenting with prototypes of solar powered systems that included fans placed inside mosquito nets, dubbed the ‘Bɔkɔɔ System’, as ‘bɔkɔɔ’ (pronunciation follows the international phonetic alphabet) is Twi for ‘I am cool’ [7], it was hypothesized that such a low energy solar net fan system could be desirable and economically affordable for a large part of the population in rural sub-Saharan African settings, and that access to such a fan system could increase bed net usage in areas with a hot climate [7, 8].

A recent trial of these simple devices was conducted in Ghana which demonstrated the potential of the ‘Bɔkɔɔ System’ to increase the use of LLINs [9]. As part of the trial a willingness to pay study was conducted at the conclusion of the trial to determine the potential for users to purchase these systems and to estimate the demand curve for a ‘Bɔkɔɔ System’ in rural Ghana, an area with limited external power availability.

## Methods

Subsequent to the completion of a cluster randomized cross over trial conducted in rural Greater Accra where participants were provided with the ‘Bɔkɔɔ System’ and household water filters as alternatives, all trial participants were invited to participate in a Becker–DeGroot–Marschak (BDM) auction to determine the mean willingness to pay for such systems and to estimate the demand curve for the systems [10]. The auction methods are described in detail below.

### Study site

The trial was conducted in rural Greater Accra, an area with low (23.2%) net use despite relatively high levels of access to LLINs (71%) [11–13]. Of African countries with a Demographic and Health Survey (DHS) in the 2012–2015 period, Ghana ranked near the bottom in net use, even among those reporting to have a net (57.5% use). Rural Greater Accra had the lowest use of all rural strata in the country [12, 13].

Southern Ghana has a humidity index (humidex) greater than 30 year round [8], indicating that this area is extremely hot and humid, which is likely to be a barrier to consistent mosquito net use. In the Greater Accra Region, the Dodowa Demographic Surveillance Site (DSS) in Shai-Osudoku District (formerly Dangme West District), specifically the villages of Apese (Abuminya) and Amanfro, were selected for the trial and subsequent auction. These villages were chosen partially because, in addition to their rural location, they lack access to the main electricity grid.

### Product

**Fig. 1:**
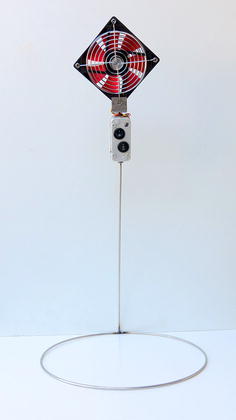
The ‘Bɔkɔɔ’ net fan

The ‘Bɔkɔɔ System’ is a simple system consisting of a variable speed fan and a light emitting diode (LED), powered by a battery attached to a small rooftop mounted solar panel via a charge controller. The fan and light console of the system is shown in Fig. 1.

### Study design and auction procedure

All eligible households, 83 of 104 total households, in the two study villages were invited to participate in the original trial. During the original trial, all participating households had solar power systems installed. Eligibility criteria for households in the trial were having more than one member and having no members who helped with the trial implementation. The trial was a three-armed cluster (household) randomised control cross-over trial. The three arms consisted of households who received net fans at the start, and were later crossed over to water filters instead of net fans, a second arm where households received water filters at the start of the trial and were later crossed over to net fans, and a third control arm which received neither intervention. All households received enough LLINs to cover all sleeping spaces in the household [9]. All participating households in the trial recieved the solar panel system and these panels were not included in the auction procedure. All participating households in the original trial were asked to participate in the BDM auction once the original trial reached completion. The auction process followed the following procedure: prior to the auction, all study households were informed of the upcoming auction and its’ basic parameters during a community information meeting, such that, if necessary, participants could plan to have money available to participate in the auction. On the day of the auction, the head of each participating household was asked to participate in the BDM auction. After providing consent to participate, they were first trained in the BDM auction procedure using a bidding exercise with candy. Once the training exercise was complete and participants demonstrated understanding of the process, the BDM auction for a net fan began. Auctions were conducted at participants’ residences.

Participants were instructed that they would make a bid for a single net fan. Next, the study participant would make a random draw of chip from a bag with a price ranging from 10 to 80 Ghanaian Cedis (~2–20 USD), in increments of 10 Cedis, marked on it. If the bid price given by the study participant was ≥ to the randomly drawn price, the study participant was expected to purchase the fan at the drawn price or to make an advance payment on the fan system which could be redeemed within 2 weeks if the participant returned with the total amount of money required for the purchase. Those participants whose bid was < the drawn price lost the auction and did not pay any amount nor did they receive a fan and light console during the auction study. All study fans which were not purchased as part of the auction were removed after the study was complete. These participants were offered an opportunity to purchase fans at a later date, though this opportunity was not disclosed until the auction was complete. Following the BDM auction, participants were also offered the opportunity to purchase additional fans at the drawn price, up to a maximum equal to the number of household members. Coupons were issued to all study participants who agreed to place down payments on the fan systems rather than make payment in full at the time of the auction. Data from the auction was analysed using logistic regression to estimate the probability of any fan purchase at a specified price and Poisson regression to determine the quantity of fans purchased relative to household occupancy at a specified price. Individual and household level determinants of bid price were analysed using linear and ordered logistic regression.

## Results

Out of 83 eligible households, 80 participated in auctions; results are summarized in Table 1. Of these auctions, in only 64 was the randomly drawn price equal to or lower than the participant bid price. In one of these 64 cases, the auction was won (the participant’s bid was greater than or equal to the randomly drawn price), but the participant refused to make a direct purchase or place an advance payment. The mean willingness to pay for a ‘Bɔkɔɔ’ net fan was 55.0 Cedis (95% C.I. 50.8–59.2). A total of 94 fans were purchased in the 80 auctions. Of these fans, 54 were bought at the auction and 40 were purchased at a later date after the participants placed a small, 10% of the balance due, advance payment on the day of the auction. Advance payment redemption was relatively low (~50%), participants placed advance payments on 85 fans, but redeemed only 40 of these within 30 days. The advance payments which were not redeemed within 60 days were not included in further analysis as doing so would positively bias the estimates of WTP. Only households who won the auction were allowed to place advance payments.

**Table 1:** Results of BDM auction. Bid and drawn prices given in Ghanaian Cedi

Outcome	N HH	N members	Avg. bid	Avg. drawn	N of fans purch.
Won auction	64	252	60.6	32.2	94
Lost auction	16	61	32.5	63.1	0
All	80	313	55.0	38.8	94

*N HH* number of households

**Fig. 2:**
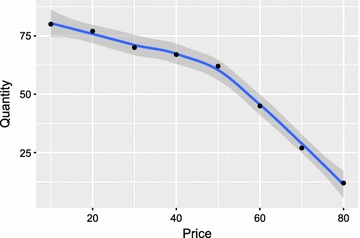
Demand for the ‘Bɔkɔɔ’ net fan vs. bid price per unit. Number of households (of 80 total) expected to purchase at least one fan system at a specified price. Loess smoothing used to estimate regression line, points are the number of bids at or above the specified price

**Fig. 3:**
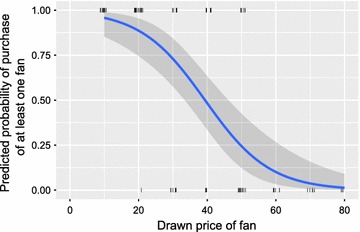
Demand for at least one ‘Bɔkɔɔ’ net fan vs. drawn price per unit. Predicted probability of purchase estimated with logistic regression.* Tick marks* shown at various points along the x-axis and at either one or zero on the y-axis represent actual data points with a small amount of random noise added in the x-direction

**Fig. 4:**
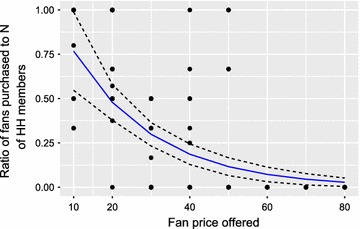
Total demand for ‘Bɔkɔɔ’ net fan vs. drawn price per unit. Slope estimated with Poisson regression, 95% C.I. for regression result shown with* dotted lines*.* N* number,* HH* household

Figure 2 shows the results of the auction as a standard demand curve with the quantity expressed as the number of households which would purchase at least one ‘Bɔkɔɔ’ fan and light console at a given price. This shows that fewer households are likely to purchase a fan with increasing price and that as the price approaches 80 Cedis, few households would purchase any. Eighty total households participated in the auction.

Figure 3 shows the predicted probability of the purchase of at least one ‘Bɔkɔɔ System’ at a given price when fit with a logistic regression. Illustrating the same principles as Fig. 2. At a price of 40 Cedis ~50% of households are expected to purchase a fan system.

To estimate the total demand at a given price from the follow up sales data we fit a Poisson regression to the total numbers of fans purchased per household. Regression results indicate that demand was price elastic, with an estimated average reduction in demand of one fan per household with each 20 Cedi (~5 USD) increase in price. Elasticity varied significantly over the price range included in the study as can be seen from the varying slopes of the regression lines in Figs. 2, 3 and 4. Results of the Poisson regression on total demand are shown in Fig. 4.

The relationship between randomization group, participants’ education level, religion, profession, number of household members, use of mosquito coils, monthly income, and sex with WTP was assessed using linear and ordered logistic regressions, but none of these variables, alone or in combination showed significant relationships with bid price for fan systems.

## Discussion

Based on a review of the literature, this is the first study to estimate the willingness to pay for a small solar powered fan and light console designed to increase comfort and provide night time light to users of bed nets. The results indicate that such a product could be sold on a local commercial market and that a majority of rural households in our study are would be willing to purchase such a system at a price of approximately 40 Ghanian Cedis. This price, equivalent to approximately 10 USD, would allow full cost recovery of manufacturing costs for such systems. These results also indicate that at lower prices (~2.5 USD) that nearly all households would purchase a ‘Bɔkɔɔ’ fan and most would purchase enough fans to allow use by all household members. These findings are also confirmed by qualitative research undertaken alongside the trial on the acceptability and use of the ‘Bɔkɔɔ System’ which showed that the fan systems were very likely to be utilized by households and that most found the fan system increased the comfort of a net user and thus made them more likely to utilize nets on hot and humid nights [14]. Fan consoles had a small LED light which may have increased their desirability to households and it was not possible to determine how much, if at all, LEDs added to the WTP for these fans. Qualitative research on this topic lends creedence to the idea that the LED lights positively influced WTP. According to Jaeger et al. “the LED light on the fan stand became the main source of light at night and positively influenced the perception of the intervention as a whole [14].”

Results of the cluster randomized controlled trial conducted alongside this study indicated that availability of these systems at the household level in combination with availability of LLINs increased the usage rates of LLINs [9]. Increased net use may improve the effectiveness of LLINs in practice. Nets that are unused provide neither personal protection from mosquito biting nor are they likely to provide significant community protection. Even if an unused LLINs is hung, the net will not act as a baited trap for mosquitoes unless a human is utilizing the net [15].

This study was only conducted in two small communities in rural Greater Accra, who had experience using the system and had a power source installed, and as such the demand estimated for the ‘Bɔkɔɔ’ net fans in this context may not translate to other areas where weather conditions, housing types, sleeping arrangements, household wealth and availability of a power supply and capital may vary. Although randomisation group was not statistically significantly associated with WTP, all households in the study area might have some exposure to the fan systems, and this may have increased WTP overall. This study utilized a BDM method for estimating WTP, these methods are generally considered to be incentive compatible and as such, are not thought to be vulnerable to strategies to ‘game’ the system by overbidding or underbidding [10]. Thus BDM methods are expected to elicit true revealed WTP, rather than a hypothetical stated WTP. In addition, because households were expected to utilize their own financial resources to purchase the ‘Bɔkɔɔ’ net fan the WTP was also contingent on the availability of household funds. Some studies offer cash upfront to households to remove this constraint—this study did not—as such it is likely to better reflect true demand in the study area. Given that the study was conducted during harvest season, households may have had more cash available during the study than at other times of the year.

## Conclusions

This study estimated the willingness to pay and potential demand for a solar powered bednet fan and light console, part of the ‘Bɔkɔɔ System’, in rural Greater Accra, Ghana. The mean WTP was approximately 55 Cedis (~13 USD). We also demonstrated demand suggesting that at a price that would support full manufacturing cost recovery a majority of households in the area would be willing to purchase at least one such system.
